# SCNS: a graphical tool for reconstructing executable regulatory networks from single-cell genomic data

**DOI:** 10.1186/s12918-018-0581-y

**Published:** 2018-05-25

**Authors:** Steven Woodhouse, Nir Piterman, Christoph M. Wintersteiger, Berthold Göttgens, Jasmin Fisher

**Affiliations:** 1 Department of Hematology, Cambridge Institute for Medical Research, University of Cambridge, Cambridge, CB2 0XY UK; 2 Wellcome Trust - Medical Research Council Cambridge Stem Cell Institute, University of Cambridge, Tennis Court Road, Cambridge, CB2 1QR UK; 3 Microsoft Research Cambridge, 21 Station Road, Cambridge, CB1 2FB UK; 4 Department of Informatics, University of Leicester, University Road, Leicester, LE1 7RH UK; 5 Department of Biochemistry, University of Cambridge, Cambridge, CB2 1QW UK

**Keywords:** Executable biology, Gene regulatory networks, Developmental biology, Single cell

## Abstract

**Background:**

Reconstruction of executable mechanistic models from single-cell gene expression data represents a powerful approach to understanding developmental and disease processes. New ambitious efforts like the Human Cell Atlas will soon lead to an explosion of data with potential for uncovering and understanding the regulatory networks which underlie the behaviour of all human cells. In order to take advantage of this data, however, there is a need for general-purpose, user-friendly and efficient computational tools that can be readily used by biologists who do not have specialist computer science knowledge.

**Results:**

The Single Cell Network Synthesis toolkit (SCNS) is a general-purpose computational tool for the reconstruction and analysis of executable models from single-cell gene expression data. Through a graphical user interface, SCNS takes single-cell qPCR or RNA-sequencing data taken across a time course, and searches for logical rules that drive transitions from early cell states towards late cell states. Because the resulting reconstructed models are executable, they can be used to make predictions about the effect of specific gene perturbations on the generation of specific lineages.

**Conclusions:**

SCNS should be of broad interest to the growing number of researchers working in single-cell genomics and will help further facilitate the generation of valuable mechanistic insights into developmental, homeostatic and disease processes.

**Electronic supplementary material:**

The online version of this article (10.1186/s12918-018-0581-y) contains supplementary material, which is available to authorized users.

## Background

Executable gene regulatory network models have been successfully built and used to obtain a better mechanistic understanding of developmental, homeostatic and diseased cellular decision making processes [1]. Executable models capture essential qualitative details of a biological process and aim to mimic the order of events and the long-term behaviour of the system. These models are amendable to the use of state space analysis [2] and model checking algorithms [3] to analyse all of the many possible executions of the model and generate new predictions that can be tested experimentally. An executable model can also be used to obtain a global dynamic picture of how the system responds to various perturbations. Successful examples of executable modelling include the Boolean network models of sea-urchin development [4, 5] and of blood stem cells [6]. However, these models were manually curated and are the result of knowledge of the structure of the network built up over decades of laboratory experimentation.

We previously introduced an approach for synthesising executable gene regulatory network models directly from single-cell gene expression time course data sets, without the need of prior knowledge of the topology of the network or a detailed specification of its behaviour, which for many systems does not exist [7]. We have now developed the Single Cell Network Synthesis tool (SCNS) into a general-purpose computational tool for the reconstruction and analysis of executable models from single-cell gene expression data. The tool is controlled via a web-based graphical interface.

**Fig. 1:**
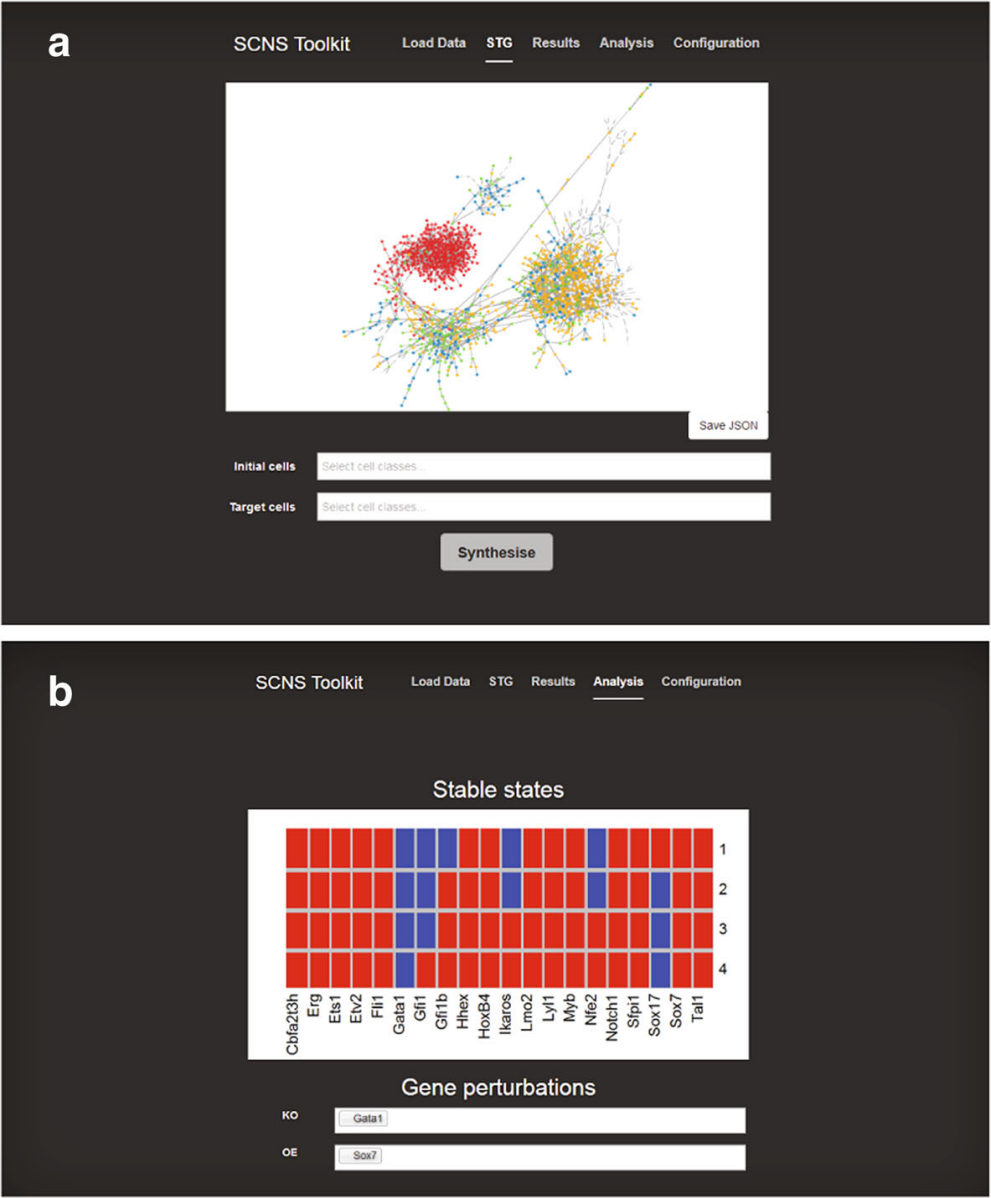
Tool overview. When SCNS is first started, the user is presented with the ‘Load Data’ page, asking them to upload a .CSV file containing their single-cell gene expression data. **a** The state transition graph page, which allows visualisation of the data, selection of initial and target cell classes, and running of synthesis. **b** The analysis page, which shows the computed stable states of the synthesised model and allows combined overexpression/knockout perturbations to be run

SCNS is a tool for understanding differentiation, developmental, or reprogramming journeys, reconstructing models from single-cell data taken across a time course. This mechanistic model explains the molecular changes underlying the biological process. SCNS takes single-cell qPCR [7–9] or RNA-sequencing data [10–14], and treats expression profiles as binary states, where a value of 1 indicates a gene is expressed and 0 indicates that it is not. It then constructs a state transition graph, where pairs of states are connected by an edge if they differ in the expression of exactly one gene (Fig. 1a). This graph is then used as the basis to reconstruct Boolean logical regulatory rules, by searching for rules that drive transitions from early cell states towards late cell states. Because the resulting models are executable, they can be used to make predictions about the effect of specific gene perturbations on the generation of specific lineages, or to suggest strategies for improving reprogramming efficiency.

An asynchronous Boolean network models a gene regulatory network by abstracting away details of transcription, translation and molecular binding reactions. Instead the status of each gene is modelled as either active (on) or inactive (off), while retaining the stochastic nature of events, and capturing the regulatory logic determining whether a gene is activated or not by Boolean update functions. Once such a model has been constructed, it can be executed, resulting in a series of single-gene changes. Asynchronous simulation of a Boolean network allows all possible interleavings of individual transitions, and therefore allows transitions to happen at different rates. The long-term behaviour over all of these possible executions can then be analysed. Since a Boolean network has a finite number of states, every execution eventually converges to either a single stable state or a cycle of states, called an attractor [2]. Stable state attractors are thought to correspond to the mature, differentiated cell types of the system [2, 15, 16]. For each gene, SCNS finds a set of Boolean rules that are compatible with the data, if more than one such compatible rule exists. The set of models resulting from taking the union of all these possible rules is then analysed. SCNS can be used to find (and display as a heatmap) stable state attractors, and to introduce every single or combined in-silico overexpression or knockout perturbation. The stable states are dynamically recomputed with the chosen perturbation and re-displayed (Fig. 1b). SCNS does not currently compute complex (loop) attractors, because of the difficulty of compactly representing these visually. However, models can be exported to the BioModelAnalyzer tool (http://biomodelanalyzer.org/); or SBMLQual format and imported into other analysis tools in order to find complex attractors.

In the last few years reconstruction of Boolean network models from biological data or behavioural specifications has become a topic of active research. For example, the RE:IN tool from Dunn et al. [17], the method of Xu et al. [18], CellNOpt [19], and the methods of Sharan and Karp [20] and Videla et al. [21]. However, the above methods cannot directly be compared to SCNS because they reconstruct models from different sources of data – prior knowledge of the gene regulatory network topology together with desired stable state specifications under wild type and/or perturbed conditions. BTR is a more comparable method, which uses single-cell expression data to refine an existing Boolean network for a system [22]. However, detailed prior knowledge in the form of an existing Boolean network for the system must exist. SingCellNet [23] uses single-cell data but only infers network topology, and the current implementation can only be applied to very small data sets.

We are aware of one tool, the Pseudotime-network-inference method of Hamey et al. [24], which is directly comparable to SCNS, as it reconstructs logical models from single-cell gene expression time course data. Pseudotime-network-inference requires a-priori information about the topology of the network, which is inferred via partial correlation. It then fits Boolean rules compatible with this topology by examining a path through a linear pseudotime ordering, rather than paths through a branching state graph. In the Example section, below, we compare the interactions predicted by SCNS to those of (partial) correlation.

## Implementation

### The SCNS algorithm

The SCNS algorithm solves the following problem:

We are given a set of variables *V* = {*v*_1_, *v*_2_, …, *v*_*n*_}, which correspond to genes measured, and an undirected state graph *S* = (*N*, *E*), where each vertex *n* ∈ *N* is uniquely labelled with a Boolean state *s* = (*x*_*1*_*, …, x*_*n*_*)*, which corresponds to an active/inactive map of the genes, and there is an edge {*s*_1_, *s*_2_} ∈ *E* iff *s*_1_ and *s*_2_ differ in the value of exactly one variable, *v*. The edge {*s*_1_, *s*_2_} is labelled with *v*. In addition, we are given a designated set *I* ⊆ *N* of initial vertices, which correspond to the measurements at an early time point, and a set *F* ⊆ *N* of final vertices, which correspond to the measurements at a final time point, along with a threshold *t*_*i*_, which, intuitively, indicates how tight a matching with the experimental data we are looking for, and a maximum number of activators *a*_*i*_ and repressors *r*_*i*_ for each variable *v*_*i*_ ∈ *V*. We would like to find an update function *u*_*i*_: {0,1}^n^ → {0,1} for each variable *v*_*i*_ ∈ *V*, such that the asynchronous Boolean network that arises from these rules satisfies the following conditions. Let *U* = {*u*_i_ | *v*_*i*_ ∈ *V*} be the set of update functions. We note that the asynchronous Boolean network defines a directed graph over a set of vertices that is larger than *N*.

1. Every final vertex *f* ∈ *F* is reachable from some initial vertex *j* ∈ I by a directed path *p*. Further, for every *v*_*i*_-labelled directed edge (*s*_*1*,_ *s*_2_) ∈ *p* we have that *u*_i_(*s*_1_) = *s*_2_(*v*_1_)
2. For every variable *v*_*i*_ ∈ *V*, let *N*_*i*_ be the set of states without an outgoing *v*_*i*_-labelled arc. For every *i* the number of states *s* ∈ *N*_*i*_ such that *u*_*i*_(*s*) = *s*(*v*_*i*_) is greater or equal to *t*_*i*_. That is, the number of edges leaving the original state space *N* is bounded.

We restrict our search to update functions of the form *f*_1_ ∧ ¬ *f*_2,_ where *f*_1 and_ *f*_*2*_ are monotone Boolean formulae (contain ∧ and ∨ gates, but no negation). The variables of *f*_1_ are *activators* of *f* and the variables of *f*_2_ are *repressors*. We look for functions with a maximum of *a*_*i*_ activators and r_*i*_ repressors.

The algorithm has three phases. We begin by building a directed graph from the given undirected state graph *S* = (*N*, *E*), by considering which of the underlying directed edges in *E* are compatible with some Boolean update function, and pruning those that are not. This phase is implemented via enumerative search, and after termination leaves us with a directed state graph *G*, which could include both directions or neither direction for a given edge.

To ensure reachability, we then construct, for each pair of initial node *i* ∈ *I* and final node *f* ∈ *F*, the shortest path from *i* to *f* in the directed graph *G* that was built in the previous phase of the algorithm. These paths can be computed via a breadth–first search.

The search for Boolean update rules compatible with these paths is then encoded as a Boolean satisfiability (SAT) problem. The update functions of each variable can be sought after separately, giving rise to reasonably sized satisfiability queries.

For full details, we refer the reader to [25, 26].

### Finding stable state attractors

To analyse together all synthesised models, we first form a combined Boolean network that makes a transition if all sub-models do. If some sub-model has a stable state attractor *s*, *s* will also be an attractor of this combined model.

Given a set of compatible update functions {*f*_*i*__1_, …, *f*_*in*_} for gene *x*_*i*_, the update function for the combined model is defined as: *f*’_*i*_ = (¬*x*_*i*_ ∧ (*f*_*i*__1_ ∧ … ∧ *f*_*in*_)) ∨ (*x*_*i*_ ∧ (*f*_*i*__1_ ∨ … ∨ *f*_*in*_))

To find a stable state *s* = (*v*_1_, …, *v*_n_) of the resulting combined Boolean network we encode the search as a Boolean satisfiability (SAT) problem: (*f*’_1_(*s*) ↔ *v*_1_) ∧ … ∧ (*f*’_n_(*s*) ↔ *v*_*n*_).

To simulate overexpression of gene *x*_*i*_, we set the target function as the constant function *f*’_i_(x) = 1. To simulate knock out, we set it to the constant function *f*’_i_(*x*) = 0.

### Software architecture and implementation

The architecture of SCNS is divided into two components: the backend and the frontend. The backend, which performs all computations necessary for the reconstruction and analysis of Boolean network models, is written in F# and makes use of the Z3 SMT solver [27].

The frontend, which implements the web-based graphical user interface and sends requests to the backend, is written in Javascript/HTML and uses the Angular library [28].

Cloud computation is implemented using the MBrace library [29, 30].

SCNS runs on Windows, Linux and macOS, but support for cloud computation is currently only supported on Windows.

### Configuration of parameters

In order to synthesise a matching Boolean network, SCNS requires the configuration of three parameters per gene. These are the maximum number of allowed activating inputs to the gene’s update function, the maximum number of allowed repressing inputs, and a threshold parameter. The threshold is a measure of how well a rule fits the data (higher is better).

In order to successfully find rules for each gene under consideration, it is often necessary to experiment with different parameter values. We recommend that one begins with loose parameters (larger number of activators and repressors, lower threshold parameter), then, once a matching logical rule has been found for a gene, to tighten these parameters (lower the number of inputs and increase the threshold) and re-run. This can be repeated until all genes have a matching rule.

The tool and source code are available at [31], under an MIT open source license.

## Results

### SCNS is controlled via a web-based graphical user interface

SCNS runs locally as a desktop application but is controlled through a web-based graphical user interface. When SCNS is first started, the user is presented with the ‘Load Data’ page, asking them to upload a .CSV file containing their single-cell gene expression data. This file should have rows corresponding to cells and columns corresponding to genes. Each entry should be a true or a false, indicating whether the cell expresses the given RNA or not. In addition, the first column, the “Class” column, should give the class of the cell. This indicates the cell type or day of measurement and is used to indicate which cell states should be considered initial states and which final states during synthesis.

The browser then automatically switches to the ‘STG’ page, where a state transition graph automatically constructed from the uploaded data is displayed (Fig. 1a). On this page the user can use two text controls to select initial and target cell classes. The ‘Synthesise’ button can then be pressed to begin synthesising Boolean network rules. The browser switches to the ‘Results’ page and Boolean update functions are displayed in a table as they become available. Reconstructed models can be exported to BioModelAnalyzer (BMA); or SBMLQual format, allowing import into analysis tools such as BMA, GINsim and BoolNet.

Once a set of models has been found, the user can navigate to the ‘Analysis’ tab to view the computed stable state attractors. They can then use two text box controls to select any single or combined overexpression or knockout perturbation. The stable states will be dynamically recomputed with the chosen perturbation and re-displayed on the page (Fig. 1b).

### SCNS synthesises asynchronous Boolean network models

SCNS uses an optimised version of the algorithm described in Woodhouse et al. [26] to synthesise an asynchronous Boolean network model from the state transition graph. SCNS is based upon viewing single-cell gene expression profiles as though they were states of an asynchronous Boolean network, and then solving the problem of reconstructing a Boolean network from its state space, as explained above. This algorithm uses a combination of enumerative search, graph reachability and Boolean satisfiability solving to extract gene regulatory network models that best match the state space data.

### SCNS supports easy deployment of computation to the cloud for performance

For data sets of up to a few thousand cells, SCNS can typically reconstruct a Boolean network model on your desktop machine within minutes. For larger data sets, SCNS can be configured to deploy computation to your Azure cloud account and parallelise operations across compute nodes.

## Example – application to pre-implantation embryonic data

**Fig. 2:**
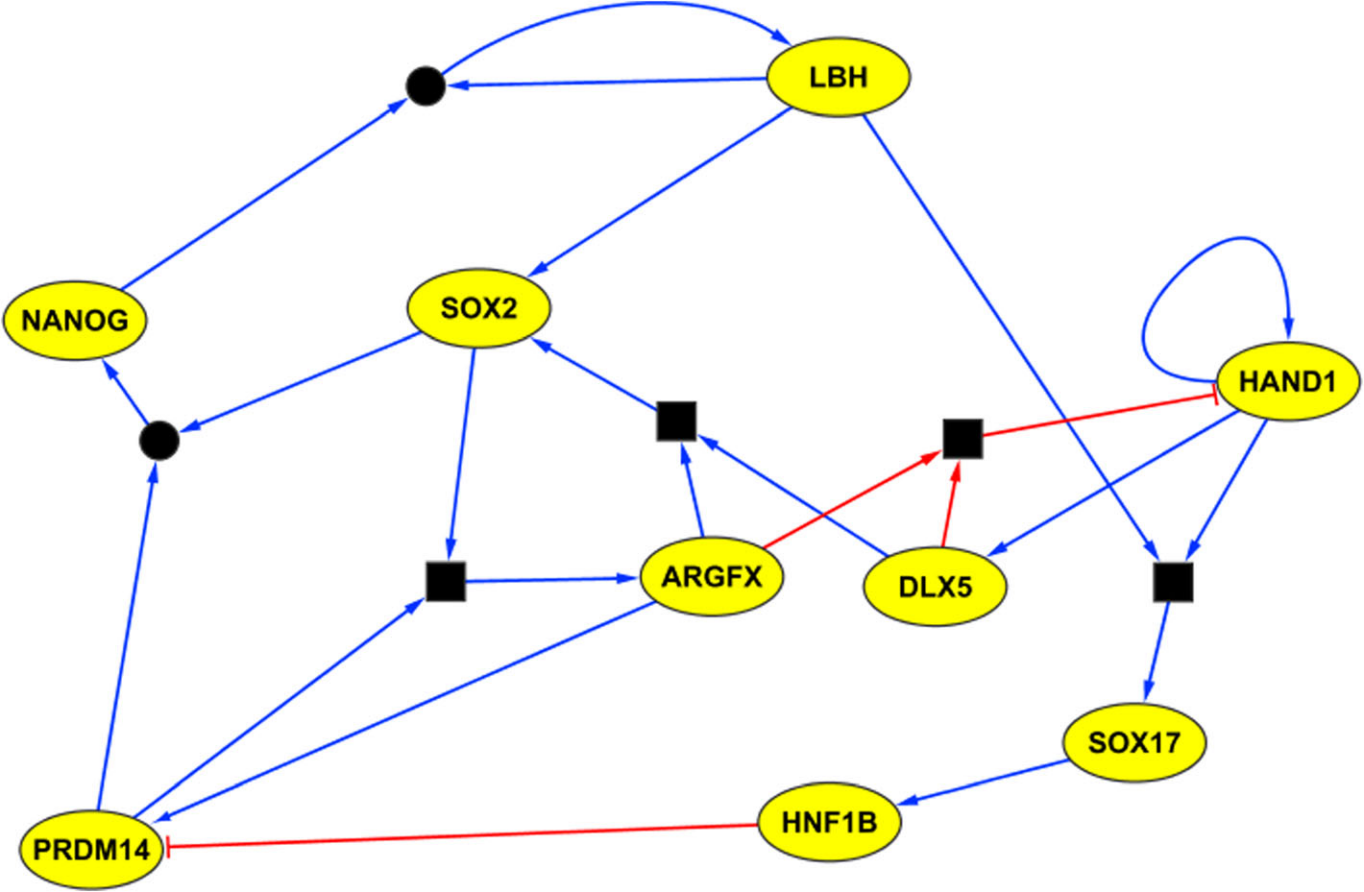
Extracted regulatory network for human preimplantation development. Blue edges indicate activation; red edges indicate repression. Square boxes represent AND operations. Circles connecting edges indicate multiple compatible update rules

We applied SCNS, using its improved optimised version of our synthesis algorithm and graphical user interface, to a recently published data set consisting of 1529 cells from early-stage human embryos [11], and extracted a connected core regulatory network for human preimplantation development (Fig. 2).

The CSV input for this example is available in the supplementary data (Additional file 1), and the parameters used to reconstruct the network are shown in Table 1. We filtered the data, selecting expression values for 20 transcription factors thought to be involved in preimplantation development. To discretise the RNA-seq data prior to uploading, we mapped zero reads to false and positive reads to true. We note that for qPCR data, false can instead correspond to measurements below the experimentally determined limit of detection. The time point or cell type of a cell is encoded as a “Class” field in the CSV file.

**Table 1:** Parameters used on example data set

Gene	Number of activators	Number of repressors	Threshold %
ARGFX	2	0	70
CDX2	3	0	70
DLX5	1	0	80
GATA2	1	0	90
GATA3	1	0	90
GATA4	1	0	80
GATA6	1	0	80
GCM1	2	0	70
HAND1	1	2	70
HNF1B	1	0	80
HNF4A	1	2	60
KLF17	1	1	80
LBH	1	0	70
NANOG	1	0	70
OVOL1	1	0	100
POU5F1	2	0	80
PRDM14	1	1	60
PRDM16	1	0	10
SOX17	2	0	70
SOX2	3	0	80

Initial cells = E3, Target cells = E7_target

### Comparison to (partial) correlation networks

We compared the network constructed by SCNS to the networks inferred via correlation and partial correlation, which are the standard approaches for inferring gene regulatory links from single-cell gene expression data [32–34], and are used as the basis of predictions of Pseudotime-network-inference [24] and RE:IN [17]. We calculated the number of directed links predicted by SCNS which are in the top 100 undirected correlating pairs predicted by correlation/partial correlation. We performed this analysis on the same 20 transcription factors that were analysed by SCNS. For this analysis we used Spearman correlation, but Pearson gives very similar results.

Of the 16 edges predicted by SCNS, 4/16 (25%) and 6/16 (38%) were not in the top 100 edges inferred by partial correlation and correlation, respectively. The fact that the majority of directed links predicted by SCNS are also (undirected) correlation relationships is perhaps to be expected, but the missing links illustrate how SCNS can detect potential gene regulatory interactions that are not apparent from correlation alone.

In particular, the negative link from DLX5 to HAND1 is not detected by correlation, as it is masked by positive correlation due to the positive link back from HAND1 to DLX5. The negative link from HNF1B to PRDM14 is also not associated with any negative correlation and the positive link from DLX5 to SOX2 is not associated with any positive correlation. Every method which relies on establishing network topology via correlation may miss such potential interactions.

## Benchmarks

To assess the efficiency of our tool we reconstructed models from four data sets: two synthetic data sets of varying sizes, the preimplantation data set from the Example section (above), and the embryonic blood data set from [7], with differing parameters. The results of these experiments are shown in Table 2. All experiments terminated within a few hours and were run on an Intel Xeon @ 3.70 GHz with 16 GB of RAM and on a single thread.

**Table 2:** Performance of SCNS on example data sets

Genes	States	Gene inputs	Run time (seconds)
11	214	2	0.5
11	214	3	4
11	214	4	11
17	1772	2	13
17	1772	3	50
17	1772	4	249
20	690	2	6
20	690	3	79
20	690	4	628
33	1448	2	1084
33	1448	3	6533
33	1448	4	Out of memory

## Conclusions

We previously applied this method to understanding the process of early blood development in the embryo, which is poorly understood due to the small number of cells present at this stage [7]. Several model predictions were validated experimentally, demonstrating that the genes HoxB4 and Sox17 directly regulate the hematopoietic factor Erg, and that forcing expression of Sox7 blocks blood development.

We have now applied SCNS, using its improved optimised version of our synthesis algorithm and graphical user interface, to a recently published data set consisting of 1529 cells from early-stage human embryos [11], and extracted a connected core regulatory network for human preimplantation development.

Reconstruction of executable mechanistic models from single-cell expression data represents a powerful approach to understanding developmental and disease processes. Understanding these networks can lead to important insights for the programmed generation of clinically-relevant cell types important for regenerative medicine, as well as into the design of molecular therapies to target cancerous cells. SCNS, as a general-purpose graphical tool for automated reconstruction and analysis of executable models from single-cell gene expression data, should be of wide interest to the growing number of researchers working in single-cell genomics.

## Availability and requirements

Project name: Single Cell Network Synthesis Toolkit

Project home page: https://github.com/swoodhouse/SCNS-GUI

Operating systems: Windows, Linux, macOS

Programming language: F# and Javascript

Other requirements: .NET or Mono. R

License: MIT

## Additional file

Additional file 1: CSV input file for human preimplantation development example data set. (CSV 85 kb)
